# Type I interferon-mediated tumor immunity and its role in immunotherapy

**DOI:** 10.1007/s00018-022-04219-z

**Published:** 2022-03-16

**Authors:** Renren Yu, Bo Zhu, Degao Chen

**Affiliations:** 1 Institute of Cancer, Xinqiao Hospital, Third Military Medical University, Chongqing, 400037 China; 2 Department of Oncology, The First Affiliated Hospital of Wenzhou Medical University, Wenzhou, 325000 China; 3 Chongqing Key Laboratory of Immunotherapy, Xinqiao Hospital, Third Military Medical University, Chongqing, 400037 China

**Keywords:** IFN-α, IFN-β, Tumor immunity, cGAS, STING, Radiation therapy, Oncolytic virotherapy

## Abstract

Immune checkpoint blockade (ICB) therapies have achieved remarkable clinical responses in patients with many different types of cancer; however, most patients who receive ICB monotherapy fail to achieve long-term responses, and some tumors become immunotherapy-resistant and even hyperprogressive. Type I interferons (IFNs) have been demonstrated to inhibit tumor growth directly and indirectly by acting upon tumor and immune cells, respectively. Furthermore, accumulating evidence indicates that endo- and exogenously enhancing type I IFNs have a synergistic effect on anti-tumor immunity. Therefore, clinical trials studying new treatment strategies that combine type I IFN inducers with ICB are currently in progress. Here, we review the cellular sources of type I IFNs and their roles in the immune regulation of the tumor microenvironment. In addition, we highlight immunotherapies based on type I IFNs and combination therapy between type I IFN inducers and ICBs.

## Introduction

**Fig. 1:**
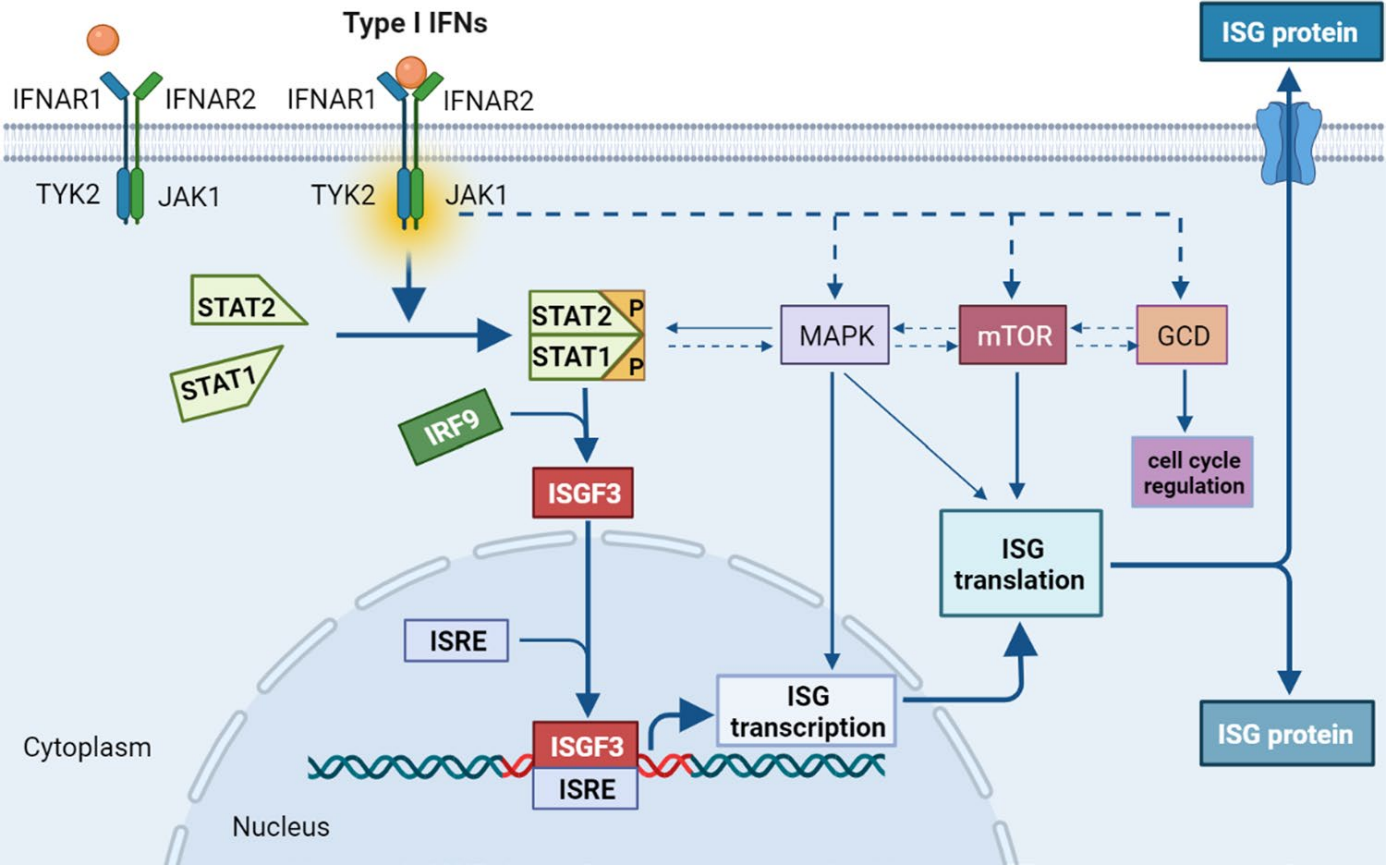
Type I IFNs signal transduction pathway. Type I IFNs bind to IFNAR1/IFNAR2 heterodimers to drive the activation of TYK2 and JAK1, which results in the accumulation of activated STAT1 and in the subsequent formation of STAT1–STAT2 heterodimers. Then, the dimerized STATs are combined with IRF9 to form ISGF3, which interacts with ISREs to induce the synthesis of various proteins from ISGs. Meanwhile, “non-classical” pathways, such as the MAPK, mTOR, and GCD pathways, are activated by type I IFNs and trigger the expression of additional ISGs

In the 1950s, in the course of their studies on the structures and properties of influenza A and other viruses, Alick Isaacs and Jean Lindenmann discovered a soluble factor that was produced by virus-infected cells and could inhibit viral infection; they named this compound “interferon” (IFN) for its capacity to “interfere” with viral replication [1, 2]. There are currently three known types of IFN: I, II, and III, which are classified by their sequence and cellular receptors [3, 4]. The type I IFN family comprises members encoded by multiple genes, including 14 highly homologous subtypes of IFN-α, IFN-β, and other lesser-known single gene products, such as IFN-ε, IFN-κ, IFN-ω, IFN-τ, IFN-δ, and IFN-ζ [3, 5–7]. The genes encoding human type I IFNs, including 13 IFN-α subtypes, as well as IFN-β, IFN-ε, IFN-κ, and IFN-ω, are found on the human chromosome 9p [3, 5, 7]. Of type I IFNs, IFN-α and IFN-β are the best understood (Fig. 1).

Both endogenous type I IFNs, which are derived from immune and tumor cells, and exogenous type I IFNs, which are produced by recombinant technology, trigger signaling cascades by interacting with their cognate transmembrane receptor, the IFN-α/β receptor 1 (IFNAR1)–IFNAR2 heterodimer [8]. Following the interaction of type I IFNs with their receptors, pre-associated tyrosine kinase 2 (TYK2) and Janus kinase 1 (JAK1) are activated, leading to the recruitment and phosphorylation of signal transducer and activator of transcription 1 (STAT1) and STAT2. Subsequently, STAT1 and STAT2 heterodimers are combined with interferon regulatory factor 9 (IRF9) to form a novel complex called IFN-stimulated gene factor 3 (ISGF3) [4, 7–11]. Then, ISGF3 translocates into the nucleus to bind IFN-stimulated response elements (ISREs) for the synthesis of various proteins from interferon-stimulated genes (ISGs) [12–14]. In addition to this classical signaling pathway, many “non-classical” pathways exist, such as the mitogen-activated protein kinase (MAPK) pathway, the mammalian target of rapamycin (mTOR) pathway, and the GCD (GCD-GTPases/cyclin-dependent kinases) pathway, which are activated by type I IFNs and trigger the expression of additional ISGs [8, 15]. This diversity of type I IFN-mediated signaling pathways may partly explain the extensive biological functions exhibited by type I IFNs.

After recombinant IFN-α2 was approved by the United States Food and Drug Administration (FDA) as the first human immunotherapeutic for cancer, type I IFNs have been widely used alone and in combination with other immunotherapeutic agents to treat solid and hematologic malignancies [11]. In this review, we outline the cell sources of type I IFNs and their immune regulation in the tumor microenvironment (TME) and discuss how the treatments that exploit type I IFN pathways could potentially be used to enhance the efficacy of immune checkpoint blockade (ICB) treatment in patients with cancer.

## Cell sources of type I IFNs in the TME

**Fig. 2:**
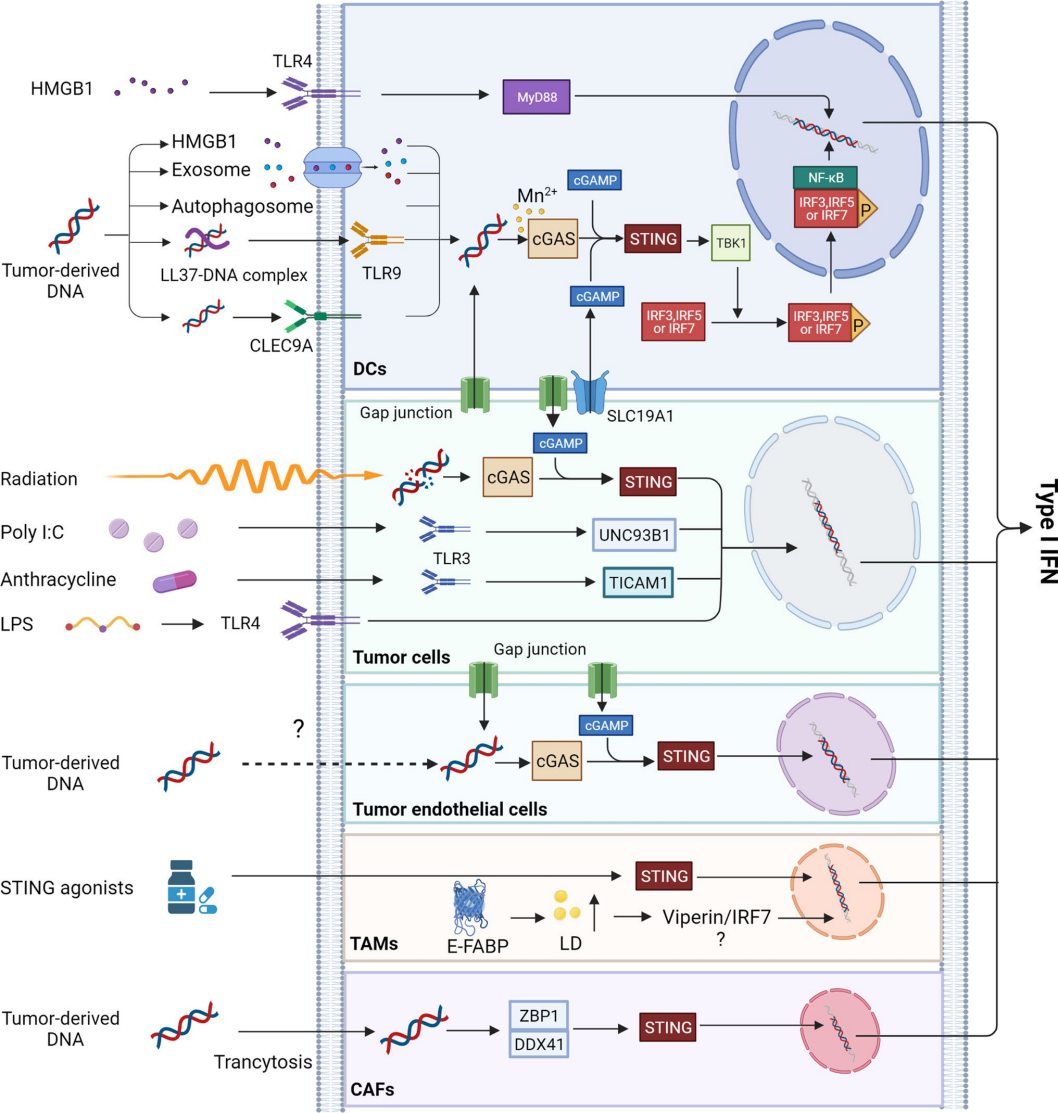
The production of type I IFNs in the TME. Tumor-derived HMGB1 induces the production of type I IFNs in DCs via the TLR4-MyD88 pathway. Tumor-derived DNA activates the cGAS/STING pathway to drive the expression of type I IFNs through chaperoning HMGB1, autophagosome, exosome, LL37, or CLEC9A into DCs. And manganese increases dsDNA binding to cGAS. Damaged DNA caused by ionizing radiation (IR) in tumor and endothelial cells triggers cGAS/STING signal to produce type I IFNs. Poly I:C induces overexpressed TLR3 to promote IFN-β production via the UNC93B1 signaling pathway in paclitaxel-resistant colon cancer (HCT-8/PTX) cells. Anthracycline induces the secretion of type I IFNs through the TLR3-TICAM1 pathway in tumor cells. TAMs expressing E-FABPs can produce high levels of IFN-β by upregulating LD. Furthermore, tumor-derived DNA enters into CAFs by transcytosis and is distinguished by ZBP1 and DDX41, then activates STING-IRF3 pathway and induces IFN-β expression

To optimize the clinical manipulation of the type I IFN system for anti-tumor therapy, it is critical to understand which cells conduce to the production of type I IFNs in the TME (Fig. 2). Although it is known that almost all cell types can produce type I IFNs in response to nucleic acids, nucleotides, or synthetic compounds[11], the primary stimulators of type I IFN production and which cells act as the primary IFN-producers in the TME have yet to be conclusively identified.

## Tumor-infiltrating dendritic cells (DCs)

Plasmacytoid dendritic cells (pDCs) are universally acknowledged to be the major producers of type I IFNs against stimulators, such as viruses, endogenous nucleic acids, and synthetic oligoribonucleotides/oligodeoxyribonucleotides [16]; however, it is not known if this paradigm holds for tumors. In solid tumors and tumor-draining lymph nodes, emerging evidence indicates that the invading host cells involved in the production of the majority of cellular IFN-β are tumor-infiltrating CD11b^+^DCs, and that the binding of tumor-derived DNA to cytosolic DNA receptors serves as the likely trigger for the activation of tumor-infiltrating DCs [17–19]. The accumulation of tumor-derived DNA in the cytoplasm is recognized by the receptor cyclic GMP-AMP (cGAMP) synthase (cGAS), which induces the formation of a second messenger, cGAMP [20–22]. Subsequently, cGAMP, in combination with the stimulator of interferon genes (STING), an adaptor protein anchored in the endoplasmic reticulum (ER), robustly initiates conformational changes and the translocation of STING from the ER to perinuclear sites, resulting in the aggregation and activation of TANK-binding kinase 1 (TBK1). In turn, activated TBK1 contributes to the phosphorylation of the interferon regulatory factor family, namely IRF3, IRF5, and IRF7, which then translocates to the nucleus and functions together with nuclear factor κB (NF-κB) to induce the abundant secretion of type I IFNs [17, 22, 23]. Recently, researchers found that manganese (Mn), a transition metal, is released from membrane-enclosed organelles upon viral infection and directly bounds to cGAS, which increases the sensitivity of the cGAS-STING pathway for double-stranded DNA (dsDNA) to produce type I IFNs [24]. Moreover, this group found that Mn could promote DC maturation and antigen presentation for antitumor immune responses through the cGAS-STING pathway and type I IFN induction [25]. Additionally, high-mobility group box 1 (HMGB1) proteins derived from dead tumor cells trigger toll-like receptor 4 (TLR4) on DCs to produce type I IFNs through myeloid differentiation factor 88 (MyD88) signaling [26].

However, the underlying mechanism by which tumor cell DNA gains entrance to the cytoplasm of DCs has not yet been established. One possible mechanism of this DNA transfer is through a medium, such as the antimicrobial peptide LL37 [27] and the C-type lectin domain-containing 9A (CLEC9A) receptor [28], that can mediate the uptake of DNA from dying tumor cells. Another possible mechanism is the chaperoning of HMGB1, autophagosome, or exosome uptake to achieve DNA transfer [18]. Gap junctions between tumor cells and DCs might be another mechanism for tumor cell DNA transfer into the cytosol of tumor-infiltrating DCs [29]. In addition, cGAMP in tumor cells, acting as a secreted immunotransmitter, can enter bystander cells through connexin-mediated channels, such as the recently discovered cGAMP transporter SLC19A1, to trigger the cGAMP-STING pathway and cause IFN-β production [30].

Although RNAs have historically been considered the principal stimuli for IFN-α and IFN-β production by activating IRF3 through retinoic acid­inducible gene I (RIG-I)-like receptors (RLRs) or toll-like receptors (such as TLR3, TLR7) during antiviral immune responses, tumor-derived RNA is only minimally stimulatory in growing tumors [18].

## Tumor cells

Tumor cells are the most abundant components in the TME and are important IFN-β producers after recognizing different pattern recognition receptors (PRRs). For instance, the contribution of overexpressed TLR3 induced by poly I:C to the production of IFN-β via the TLR3-UNC93B1 signaling pathway was observed in paclitaxel-resistant colon cancer (HCT-8/PTX) cells[31]. Anthracycline-dependent production of type I IFNs was also demonstrated to be produced by tumor cells following activation of the TLR3-TICAM1 signaling pathway [32]. B16 murine melanoma cells stimulated with TLR4 agonists, such as lipopolysaccharide (LPS), contributed to IFN-β induction [33]. Furthermore, evidence suggests that the degradation of cytosolic dsDNA induced by radiation is the trigger for type I IFN secretion in tumor cells; the tumor cell-intrinsic activation of type-I IFNs was also confirmed to be dependent on the cGAS-STING pathway [34, 35]. In addition, aside from being produced directly through the cGAS-STING pathway, IFN-β production by tumor cells might occur indirectly through the existing gap junctions, where cGAMP transfers from DCs to the tumor cells, thus inducing the transcription of IFN-β genes [17, 36].

## Tumor endothelial cells

Tumor vascular research has mainly focused on tumor endothelial cells. In one experiment, researchers unexpectedly found that tumor endothelial cells, not DCs, are the main IFN-β producers upon spontaneous and enforced STING activation [37]. The possible mechanisms are their higher capacity to produce IFN-β in response to STING activation and their relative abundance in the TME compared with tumor-infiltrating immune cells [37]. In addition, no IFN-α was found in the TME, most likely due to the weak capacity of endothelial cells to produce IFN-α upon STING activation or the relative absence of IRF7, upon which IFN-α expression depends [37, 38]. Similarly, tumor DNA and tumor-derived cGAMP might act as stimuli for IFN-β production in endothelial cells via the signaling cascade involved in the cGAS-STING pathway. However, the mechanism by which they transfer into intracellular endothelial cells remains unclear. As mentioned above, gap junctions, connexin-mediated channels, and phagocytosis are possible mechanisms [39].

## Tumor-associated macrophages (TAMs)

Macrophages are important target cells for viral infection and the mainly infiltrated immune cells in many solid TMEs; however, macrophage-produced type I IFNs are rarely observed in the TME. Interestingly, after stimulation with STING agonists, such as ML-RR-S2 CDA and DMXAA, IFN-β expression was observed in many cell types, such as DCs, bone marrow-derived macrophages, T lymphocytes from naive mice, and mouse embryonic fibroblasts, but not in B16 murine melanoma cells [40]. Furthermore, researchers found that TAMs that were sorted from pre-established B16 tumors expressed the highest IFN-β after being treated with STING agonists compared to that of DCs, T cells, and endothelial cells [40]. This study suggests that TAMs might be a type I IFN source in the TME. This is consistent with a previous study which found that TAMs expressing epidermal fatty acid-binding proteins (E-FABP) can produce high levels of IFN-β by upregulating lipid droplet (LD) formation [41].

## Cancer-associated fibroblasts (CAFs)

A recent study found that the expression of IFN-β genes can be induced by cytoplasmic DNA sensors sensing aberrant DNA in CAFs. Following transcytosis of tumor cell DNA into CAFs, cytoplasmic DNA sensors (ZBP1 and DDX41) mechanically detect aberrant DNA, which then contributes to the activation of the STING-IRF3 pathway, resulting in the expression of IFN-β and other cytokines [42]. In addition, CAFs isolated from patients with breast cancer are capable of producing IFN-β upon stimulation of DNA fragments released by apoptotic cells [43]. In a recent study, breast CAFs were also shown to produce IFN-β after co-culture with the human breast epithelial cell line MDA-MB-231 [44].

## The effect of type I IFNs on anti-tumor immunity

**Fig. 3:**
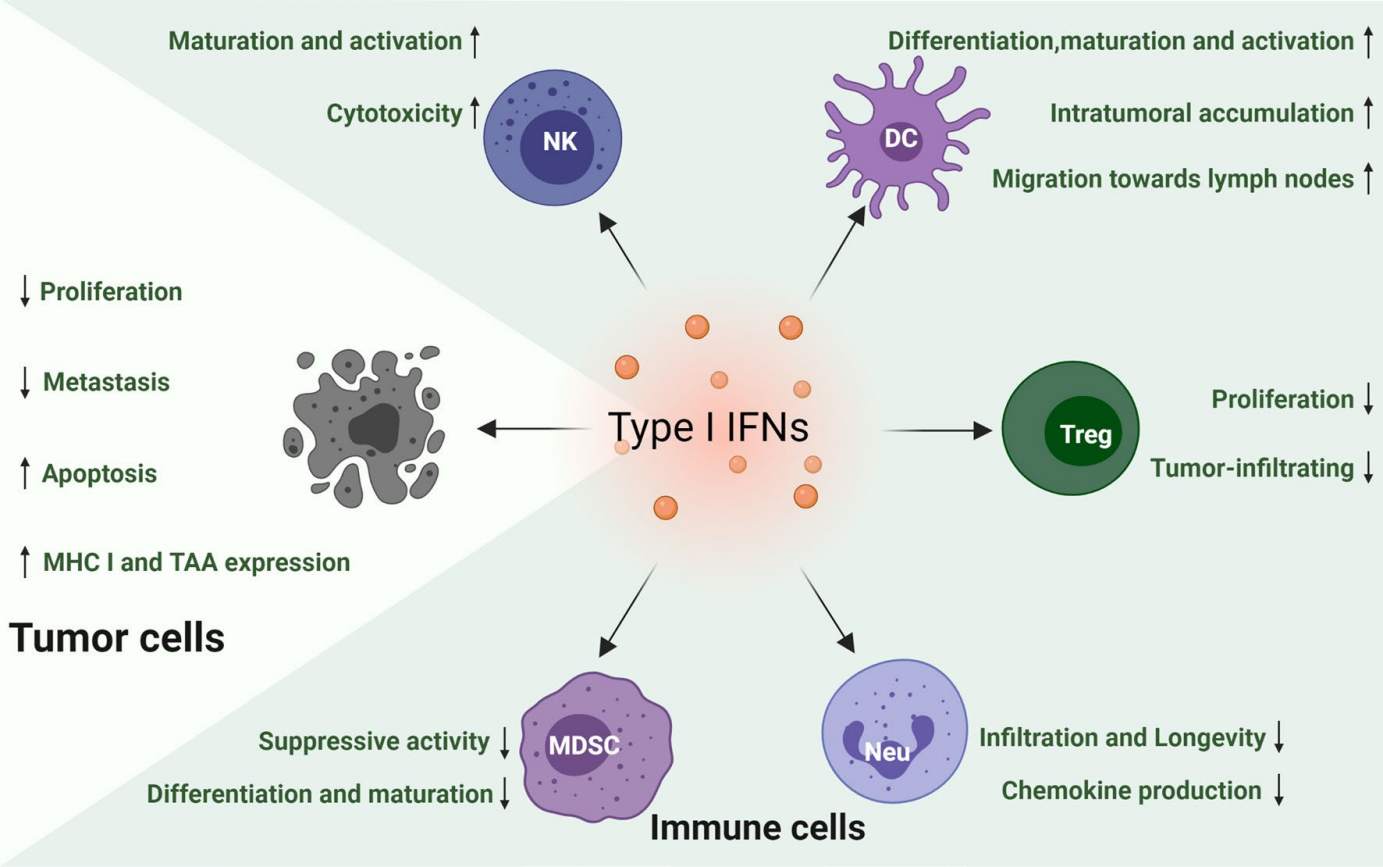
The effects of type I IFNs in the TME. Type I IFNs induce tumor cell apoptosis and inhibit tumor cell proliferation and metastasis. In addition, type I IFNs upregulate TAA and MHC I expression in tumor cells. Type I IFNs are essential for NK cell maturation and activation. Moreover, type I IFNs increase NK cell cytotoxicity. Type I IFNs promote DC differentiation, maturation, and migration into lymph nodes to activate CD8^+^ T cells. Moreover, type I IFNs increase DC intratumoral accumulation. Type I IFNs reduce Treg infiltration into tumor and Treg proliferation. Type I IFNs inhibit neutrophil infiltration, longevity, and chemokine production. In addition, type I IFNs decrease differentiation and maturation of MDSCs, and block their suppressive activity on CD8^+^ T cells

Ever since type I IFNs were first demonstrated to possess therapeutic anti-tumor potential over 50 years ago [45], an increasing number of researchers have studied the prominent role that type I IFNs play in tumor immune surveillance. It is widely accepted that type I IFNs can affect tumor cell growth, proliferation, differentiation, migration, apoptosis, and other functions via cytotoxic, cytostatic, and antiangiogenic effects [46]. In addition, type I IFNs upregulate major histocompatibility complex class I (MHC I) proteins and enhance tumor-associated antigen (TAA) expression, resulting in both increased recognition and uptake of TAA by antigen-presenting cells (APC) and antigen presentation to cytotoxic T cells (CD8^+^ T) [3, 9]. However, a recent study found that the activation of type I IFN signaling induced by radiation can help tumor cells avoid CD8^+^ T cell-mediated killing through the regulation of the serine proteinase inhibitor Serpinb9. This result might suggest a mechanism by which tumor cells develop resistance to antitumor immunity after radiation as well as potential targets for intervention to improve antitumor effects [47]. Thus, type I IFNs may play a dual role in tumor cells for tumor immunity. An overview of the effects of type I IFNs on different target cells in the TME is presented in Fig. 3.

## Dendritic cells

The predominant role of DCs in antitumor immune responses is to uptake and present TAAs to tumor-specific CD8^+^ T lymphocytes. In this process, type I IFNs exert multiple effects on DCs, most likely by stimulating DC differentiation and maturation and up-regulating the activity of DCs via cross-presentation of TAAs to CD8^+^ T cells [3]. Notably, the migration of professional APCs from the tumor site to the lymph nodes is a key prerequisite for immune response initiation. Experiments have shown that type I IFNs are required for DCs to promote their migration toward lymph nodes [9, 19]. Moreover, although it is clear that type I IFNs can induce intratumoral accumulation of CD8α^+^ DCs, the detailed mechanism by which this occurs remains unclear [19]. Patients with various malignancies, particularly renal cell carcinoma or melanoma, who received DC vaccines combined with or without recombinant IFN-α2, have shown encouraging objective responses or sometimes long-term patient survival [4, 48]. Nevertheless, more preclinical and clinical studies are still needed to confirm the safety and effectiveness of type I IFN-stimulated DC vaccines.

## Natural killer cells

Natural killer (NK) cells, which are generally the first line of defense against pathogenic infection and tumors, are dependent on type I IFNs for maturation, activation, and homeostasis in the TME [49, 50]. NK cells are significantly decreased and exert heavily impaired cytotoxic capacity in IFNAR1 and IFNAR2-deficient mice compared with their wild-type counterparts, confirming the essential role of type I IFNs in NK cell activation [51]. In addition, NK cells with TYK2 and STAT1 deficiency, the downstream signaling components of the type I IFN pathway, display impaired cytotoxic function against tumor cells [51]. Moreover, NK cells can be primed by cytokines derived from other immune cells; these immune cells are activated via essential signals provided by type I IFNs. For instance, IL-15, the master cytokine that promotes NK cell maturation, proliferation, and function, can be produced by DCs in the presence of type I IFN [51]. To enhance NK cell cytotoxicity to tumor cells, a human IFN-α gene-modified NK cell line was established. The increased cytolytic activity in the NK cell line showed an upregulation of perforin, granzyme B, and Fas ligand as well as the secretion of cytokines, like tumor necrosis factor-α and IFN-γ, in vitro and in xenograft tumor models [51, 52]. Accordingly, a significant increase of circulating CD56^bright^ NK cells that produced increased levels of IFN-γ has been observed during IFN-α treatment in patients with chronic myeloproliferative neoplasms [53].

## Neutrophils

Due to their diversity and plasticity in the TME, neutrophils act as a “double-edged sword” that can mediate both tumor-promoting and antitumor effects [54]. Type I IFNs induce phenotypic and functional changes in neutrophils and tend to restrain tumor progression. Studies have shown that IFN-β interferes with the accumulation of neutrophils by suppressing the expression of the C-X-C motif chemokine receptors, CXCR2 and CXCR4, and by blocking their ligands, CXCL1, CXCL2, or CXCL12, in tumor cells [55, 56]. In addition, neutrophils exhibit decreased longevity in response to endogenous IFN-β. This might be due to the ability of IFN-β to (1) induce high cytotoxic reactive oxygen species (ROS) production by neutrophils [57], (2) modulate the expression of the proapoptotic Bcl-2 protein, Bax, and its anti-apoptotic counterpart, BCL-xL, in neutrophils into a proapoptotic ratio, (3) upregulate the IFN-β-dependent death receptor, Fas, on neutrophils, or (4) decrease the expression of granulocyte colony-stimulating factor, G-CSF, in neutrophils [55, 58]. In addition, type I IFNs have been shown to suppress some pro-angiogenic chemokines, such as vascular endothelial growth factor (VEGF), neutrophil-derived matrix metallopeptidase 9 (MMP9, which degrades the extracellular matrix), and CXCLs (CXCL1, CXCL2, CXCL3, CXCL5, CXCL6, and CXCL8, which are required for direct activation of endothelial cells). This serves as an antiangiogenic mechanism during tumorigenesis [56]. Moreover, although high amounts of ROS are toxic to endothelial cells, IFN-dependent ROS production by neutrophils can also exhibit antiangiogenic properties [55]. In the context of metastatic processes, type I IFNs play a crucial role in elevating the plasma levels of G-CSF and increasing the expression of CXCR2 on neutrophils [58, 59]. In summary, type I IFNs reduce the infiltration, longevity, and chemokine production of neutrophils to mediate antitumor activity [60]. Based on these findings, researchers have analyzed neutrophil characteristics in the human system and found that patients with melanoma undergoing adjuvant type I IFN therapy have lower neutrophil counts and upregulation of co-stimulatory molecules, like ICAM1, compared with the untreated control group [57, 60].

## Regulatory T (Treg) cells

Treg cells are dedicated to maintaining immune homeostasis in the host, which limits the antitumor immune response; consequently, they are considered a target for immunotherapy in the TME [61]. The suppressive effect of type I IFNs on Tregs in the TME has been extensively demonstrated. In a CT26 colon cancer model, intratumoral IFN-α gene transduction significantly reduced the frequency of Treg cells. Furthermore, IL-6, a Treg-inhibitory cytokine, was produced by intratumoral CD11c^+^ cells in response to IFN-α stimulation. In addition, IFN-α-mediated IL-6 leads to the trans-differentiation of Tregs into Th17 cells, which might partly explain the reduction in Tregs [62]. Bacher et al. indicated that IFN-α eliminates the suppressive function of Treg cells through a pathway that involves the stimulation of MEK/ERK-mediated phosphodiesterase 4 (PDE4) activation and the consequent depletion of cyclic AMP (cAMP) [63]. In addition to directly inhibiting Treg proliferation and function, type I IFNs can indirectly limit the recruitment of Treg cells to the TME by blocking CCL22, a Treg-attracting chemokine that is extensively expressed in many tumors and is beneficial to the intratumoral accumulation of Treg cells [64]. Similarly, when CCL17, another Treg-attracting chemokine expressed on CT26 cells, was blocked by IFN-α, tumor-infiltrating Treg cells decreased and CT26-specific CD8^+^ T cells increased [65]. In human breast cancer, tumor-associated pDCs are highly repressed due to their ability to produce IFN-α. This defect in IFN-α production strongly promotes the infiltration and expansion of Treg cells in the TME and leads to tumor progression and poor survival [66].

## Myeloid-derived suppressor cells

Similarly, myeloid-derived suppressor cells (MDSCs), which play a significant role in tumor-associated immunosuppression and are known to hamper successful immunotherapies in tumor-bearing hosts and patients with cancer [67], have been shown to be influenced by type I IFNs. For instance, in a C26 colon carcinoma model, the in vivo short-term application of recombinant IFN-α disturbed the differentiation and maturation of MDSCs and blocked their suppressive function on T cell proliferation [68]. Furthermore, treating tumor-bearing mice with the TLR3 and MDA-5 ligand poly I:C reduced the suppressive activity of MDSCs and induced the production of high amounts of type I IFNs [69]. In addition, a study found that autocrine IFN-α/β from the TME upregulates TRAIL expression on activated T cells, which elicits MDSC apoptosis through the TRAIL–DR5 pathway. Furthermore, they found that using neutralizing IFNAR1 abolishes the type I IFN-induced MDSC apoptosis [70].

## Immunotherapies based on type I IFNs

**Fig. 4:**
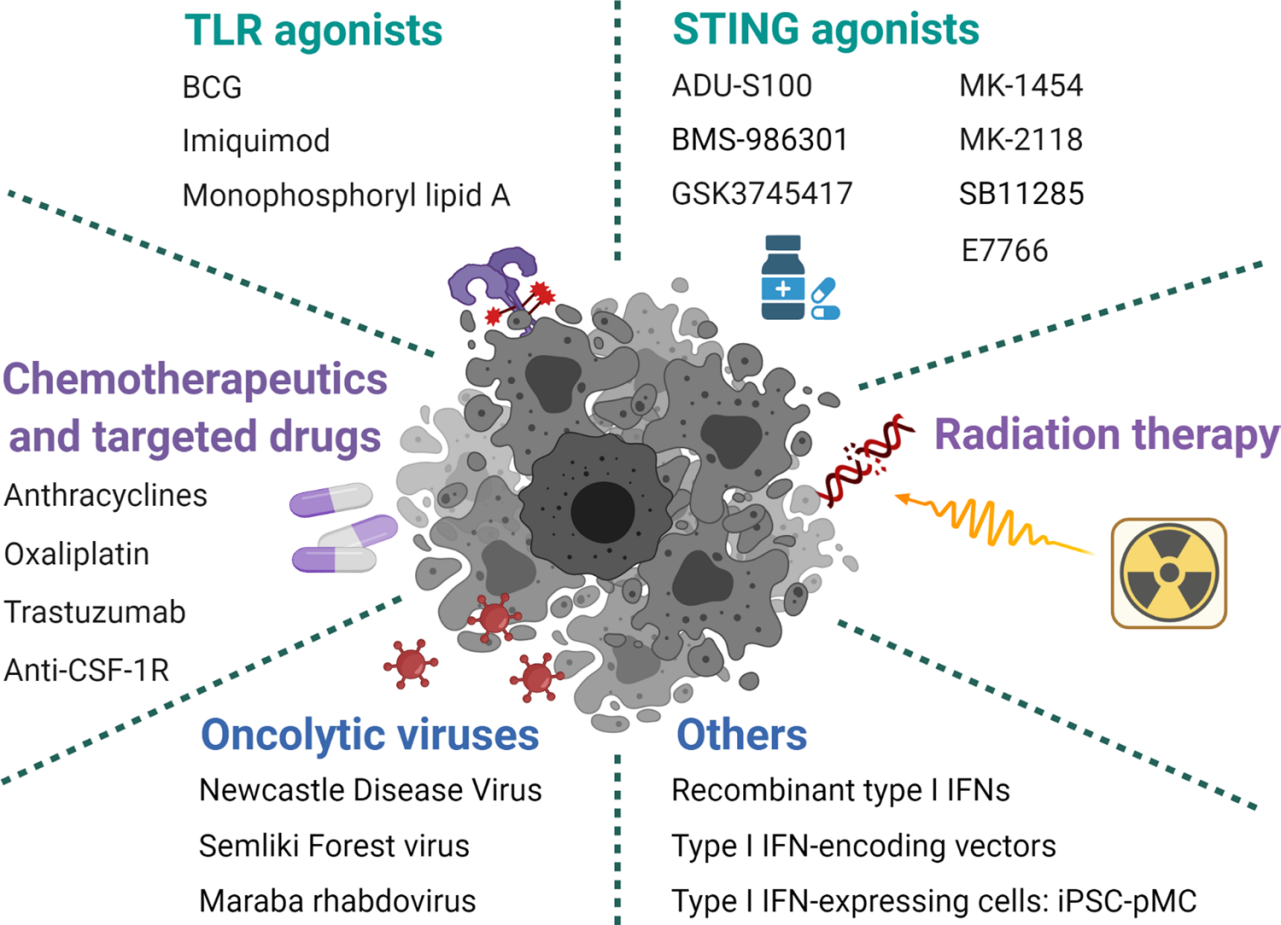
Immunotherapies based on type I IFNs. Type I IFN inducers, including TLR agonists, STING agonists, chemotherapeutics and targeted drugs, radiation therapy, oncolytic viruses, and others, are undergoing preclinical and clinical trials

It is widely accepted that type I IFNs have a great impact on both tumor inhibition and stimulating antitumor immune responses, while the systemic administration of type I IFNs is accompanied by many adverse outcomes, including fatigue, nausea, anorexia, flu­like symptoms, dizziness, hepatotoxicity, severe depression, leukopenia, and possibly the expression of immunosuppressive enzyme indoleamine 2,3-dioxygenase 1 (IDO1) [71, 72]. Therefore, attempts have been made to target the delivery of type I IFNs to the TME. As an anti-tumor strategy, treatment with type I IFN agonists is usually preferred over the use of recombinant type I IFN (Fig. 4).

## TLR agonists

TLR agonists, including poly A:U, poly I:C, poly I:C plus polylysine (poly(ICLC)), CpG adjuvant, Bacillus Calmette–Guérin (BCG), monophosphoryl lipid A, imiquimod (R837), resiquimod (R848), and motolimod (VTX-2337), are all potent type I IFN inducers [3, 73–76]. Poly I:C was recently reported to reinforce the potency of cytotoxic chemotherapeutics in paclitaxel-resistant colon tumor cell lines through the TLR3-UNC93B1-IFN-β signaling cascade [31]. In the preclinical studies, researchers mainly focused on the delivery efficiency of TLR agonists into the tumor site; intratumoral injection or nanoparticle-conjugate of TLR7/8 agonists were widely studied to overcome lowering the local drug dose in tumors [77–80]. The only TLR agonists currently approved for use by the FDA for the treatment of human patients with cancer are BCG, monophosphoryl lipid A, and imiquimod. Trials using poly A:U, poly I:C, or poly(ICLC) have demonstrated clinical benefits in several tumors (NCT00694551/ NCT00058123/NCT01188096) [3], but the use of resiquimod, motolimod, and other TLR7/TLR8 agonists as immunostimulatory agents in patients with cancer have shown disappointing results in recent clinical tests [76, 81, 82].

## STING agonists

Since STING stimulation results in type I IFN production, many cyclic dinucleotides (CDNs) have been synthesized to stimulate the STING signal. DMXAA, also known as ASA404 or vadimezan, is a strong STING agonist without significant local or systemic toxicity and was shown to have anti-tumor immunity in mouse models. In a previous study, DMXAA showed anti-angiogenic effects [83], and recent reports found that STING activation by DMXAA reduced bone cancer pain and local tumor burden [84], and promoted CAR T cell trafficking and persistence in breast cancer [85]. Although it had various beneficial effects in preclinical models, DMXAA was ineffective when combined with platinum-based chemotherapy in a phase 3 efficacy trial in human patients with advanced non-small-cell lung cancer (NSCLC) [86, 87]. Other STING agonists include ADU-S100, BMS-986301, GSK3745417, MK-1454, MK-2118, SB11285, and E7766. Among these, ADU-S100 is being tested in combination with ICBs. BMS-986301, GSK3745417, MK-1454, MK-2118, and SB11285 are being tested as standalone immunotherapeutic interventions and/or in combination with ICBs. E7766 is being tested as a standalone immunotherapeutic intervention (NCT04144140) [87].

Since intratumoral administration of CDNs notably induced STING activation, resulting in the cytotoxicity of many cell types in TME and the production of systemic inflammatory cytokines, liposomal nanoparticle-delivered or extracellular vesicle loaded STING agonists have been created. Liposomal cGAMP-NPs induced type I IFN production through STING stimulation and suppressed tumor growth by reprograming the TME. Moreover, liposomal cGAMP-NPs showed a synergistic effect with ICBs in a triple-negative breast cancer (TNBC) mice model [88]. ExoSTING, an engineered extracellular vesicle loaded with CDN, showed an enhanced therapeutic effect compared to free CDN [89]. Furthermore, the first-in-human study of ExoSTING in advanced/metastatic, recurrent, injectable solid tumors has been initiated (NCT04592484). Other STING agonists, including CRD-100 [90] and GSK532 [91], have shown potential anti-tumor immune responses and are under clinical investigation.

In addition to these direct type I IFN agonists, other “indirect” type I IFN agonists, such as chemotherapeutical and targeted drugs, radiation therapy (RT), and oncolytic viruses (OVs), are possible agents that can be used to promote type I IFN signaling.

## Chemotherapeutics and targeted drugs

Experiments in mouse models have shown that IFNAR1 expression on tumor cells and not host cells is required for the satisfactory efficacy of anthracycline-based therapy [92, 93]. Some chemotherapeutics, such as anthracyclines, promote the activation of TLR3 in mouse and human tumor cells, leading to the secretion of type I IFNs, which then activate an autocrine or paracrine IFNAR­dependent circuit that drives the expression of some ISGs [32]. Similarly, anthracyclines and oxaliplatin can induce tumor cell death via the release of HMGB1, which is recognized by TLR4 expressed on DCs and subsequently activates MyD88, resulting in type I IFN production [26]. Trastuzumab, the main treatment for human epidermal growth factor receptor 2 (HER2/ErbB-2)-positive breast cancer, has been proposed to be mechanistically dependent on the release of type I and type II IFNs [92]. Additionally, anti-colony-stimulating factor-1 receptor (anti-CSF-1R), which depletes the majority of F4/80^+^ TAMs, induces intratumoral type I IFN signaling, sensitizing tumors to cisplatin, thus improving its therapeutic effect [94]. Moreover, studies have suggested that the cisplatin + anti-CSF-1R synergistic therapeutic efficacy against tumors can be further enhanced by targeting neutrophil-dependent immunosuppression, which is critical for an antitumor immune response during cisplatin treatment [94].

## Radiation therapy

Local radiotherapy serves as a highly targeted, effective therapy for tumors due to its ability to induce lethal DNA damage and direct cell death and/or senescence. In this context, studies have shown that ablative RT increases intratumoral production of IFN-β, and the antitumor effect of RT is dependent on the production of type I IFNs [95]. Furthermore, a recent study showed that both the cGAS/STING-dependent DNA-sensing pathway and the MAVS-dependent RNA-sensing pathway are crucial for type I IFN signaling induced by radiation alone, or radiation plus ataxia-telangiectasia-mutated (ATM) and Rad3-related protein kinase inhibitor (ATRi). This suggests a mechanism by which radiation induces cell-intrinsic type I IFN signaling pathways and cytosolic nucleic acid-sensing pathways [96]. Additionally, in combination with intratumoral injection of the TLR9 agonist SD-101, RT exhibits a type I IFN-induced antitumor effect in patients (NCT02266147) [97].

## Oncolytic virotherapy

Oncolytic virotherapy is a promising cancer therapy in which replicated viruses are used to infect and replicate in growing tumor cells, leading to their lysis. In addition to their direct oncolytic ability, OVs can cause local and contained infections that activate the antitumor immune response via ectopic expression of inflammatory molecules.

T-VEC (Talimogene laherparepvec) was designed from Herpes simplex virus type 1 (HSV-1) to produce granulocyte–macrophage colony-stimulating factor (GM-CSF) and is the first approved OV by the FDA to treat surgically unresectable skin and lymph node lesions in patients with advanced melanoma [98]. Using a single-cell RNA sequence, a recent study reported that T-VEC treatment in patients with primary cutaneous B cell lymphoma induced IFN-α/β signaling and early NK and DC infiltration, which further caused cytotoxic T cell enrichment and a decrease in Treg [99]. Oncolysis by PVSRIPO, the recombinant poliovirus/rhinovirus chimera, releases the proteome of cancer cells and induces type I IFN-dominant responses in DCs, resulting in antitumor immunity [99]. In a phase 1 trial of recurrent glioblastoma, intratumoral delivery of PVSRIPO confirmed the absence of neurovirulent potential and revealed durable radiographic responses [100]. Further trials in breast cancer (NCT03564782) and melanoma (NCT03712358) are being conducted. Furthermore, a recent report found that PVSRIPO-mediated antitumor immunotherapy depended on type-I/III IFN from macrophages and DCs in the TME, but was independent of tumor cell lysis [101]. This study further supports the wide-ranging anti-tumor immune response of OVs in the TME. Local intratumor therapy of B16 melanoma with oncolytic Newcastle Disease Virus (NDV), an avian paramyxovirus with robust type I IFN-inducing and oncolytic properties, promotes lymphocyte infiltration and anti-tumor immunity in distant tumors without distant virus spread [102]. MEDI9253, a recombinant NDV encoding IL-12, has been tested in combination with ICBs in a clinical trial (NCT04613492). Similarly, the therapeutic activity of an IL-­12-­encoding variant of the Semliki Forest virus (SFV-IL12) strongly relies on a vector-induced IFN-I response that stimulates the IPS-1- and Trif-dependent pathways [103]. Furthermore, Vvax001, an RNA replicon vaccine based on SFV, has been demonstrated to be safe and induced immune responses in all participants with HPV-induced cancers [104]. Moreover, the antitumor immunity of the Maraba rhabdovirus is greatly impaired when IFNAR1 is blocked in mouse tumor models, suggesting the Maraba virus is dependent on the type I IFN-mediated anti-tumor immune response [105]. Moreover, a Maraba-based OV, MAGE-A3, is currently being tested in phase 1/2 studies (NCT02285816/NCT02879760). A recent study suggested that OVs can be co-administered with antigenic peptides in personalized anti-cancer vaccines to target patient-specific mutations [106]. However, even though OVs have been approved by the FDA and achieved certain efficacy in some patients, the resulting increase in type I IFNs can be problematic. NDV-induced type I IFN increases PD-L1 expression in the TME and mediates the resistance to immunotherapy; however, intratumoral NDV in combination with ICBs expands its therapeutic efficacy [107]. Moreover, combining adoptive cell therapy with OVs induced the autoimmune side effect of type I IFN [108] and OVs drove type I IFN to restrict CAR T cell therapy [109]. Thus, combination therapy with OVs needs to be specifically optimized. Importantly, IFNs exhibit antiviral effects, and some tumors that respond well to OV therapy often have IFN pathway defects, as these defects provide an advantage to tumors; however, this attribute makes them particularly vulnerable to viral infections and is, therefore, an intriguing prospect for OV therapy [110].

Similarly, the success of conventional chemotherapeutics, RT, and OVs, to some extent, is dependent on type I IFN signaling. In addition to these therapies, several strategies have been developed to selectively activate type I IFN systems within tumor sites. These include recombinant type I IFNs, type I IFN-expressing cells, and type I IFN-encoding vectors [72]. For instance, the induced pluripotent stem cell (iPSC)-derived proliferating myeloid cell (iPSC-pMC), a gene-modified cell, was engineered to specifically produce IFN-α; its local administration may also promote host XCR1^+^ DCs to enhance CD8^+^ T cell activation, resulting in remarkably reproducible and effective antitumor immunity without clear side effects. Furthermore, the combination of IFN-α-iPSC-pMC with ICBs increases perforin^+^CD8^+^ T cell accumulation, thereby causing a long-lasting antitumor immune response [111].

## Type I IFNs and ICBs

Despite the unprecedented clinical success of ICBs, particularly the PD-1/PD-L1 blockade, only a limited proportion of patients respond well to ICBs employed as a monotherapy treatment owing to the establishment of primary, adaptive, and acquired resistance [112]. Consequently, the development of combination therapies that promote tumor suppression has sparked the interest of researchers. ICB combination partners that have been approved by the FDA have been found to benefit a significant number of patients with cancer after combination therapies [113]. Among the many different combinatorial regimens, many ICB combination partners in development are based on type I IFNs (Table 1–4). Nonetheless, some clinical trials combination type I IFN inducers with ICBs did not acquire robust antitumor effects. Recently, report indicated that sustained type I IFN was observed in anti-PD-1 resistant tumors. PD-L1 and NOS2 expression in both tumor and DCs were induced by the sustained IFN-β transcription, and NOS2 inhibition maintained long-term control of tumors with anti-PD-1 treatment [114]. These studies suggested that the combination type I IFN inducers and ICBs should be used in specific tumor scenarios.

**Table 1:** TLR agonists in combination with ICBs

TLR agonist	ICB	Cancer type	Trial NO	Phase	Status	Refs.
TLR2/4						
BCG	Atezolizumab	High-Risk BCG naïve Non-muscle Invasive Bladder Cancer	NCT04134000	1	Recruiting	
	Atezolizumab	BCG-naive High-risk Non-muscle Invasive Bladder Cancer	NCT03799835	3	Recruiting	
	Pembrolizumab	High-Risk Non-Muscle Invasive Bladder Cancer	NCT03711032	3	Recruiting	
	Durvalumab	Non-muscle-invasive Bladder Cancer	NCT03528694	3	Active, not recruiting	
	Nivolumab	High-Risk Non-Muscle Invasive Bladder Cancer	NCT04149574	3	Recruiting	
	Tislelizumab	High-Risk Non-Muscle Invasive Bladder Cancer	NCT04922047	1/2	Recruiting	
TLR3						
Poly I:C	PD-1 mAb	Unresectable Hepatocellular Carcinoma	NCT03732547	2	Recruiting	
Poly ICLC	Pembrolizumab	Mismatch Repair Proficient Colon Cancer	NCT02834052	1/2	Recruiting	
	Pembrolizumab	Relapsing Glioblastoma	NCT03665545	1/2	Recruiting	
	Durvalumab and Tremelimumab	Advanced, Measurable, Biopsy-accessible Cancers	NCT02643303	1/2	Recruiting	
BO-112	Pembrolizumab	PD-1/PD-L1 Refractory Liver Cancer	NCT04777708	1	Not yet recruiting	
	Pembrolizumab	Colorectal or Gastric/Gastroesophageal Junction Cancer With Liver Metastasis	NCT04508140	2	Recruiting	
	Pembrolizumab	Unresectable Malignant Melanoma	NCT04570332	2	Recruiting	
	Nivolumab	Before Surgery of Resectable Soft Tissue Sarcoma	NCT04420975	1	Recruiting	
TLR7/8						
TransCon TLR7/8 Agonist	Pembrolizumab	Advanced or Metastatic Solid Tumors	NCT04799054	1/2	Recruiting	
BDB001	Atezolizumab	Advanced Solid Tumors	NCT04196530	1	Active, not recruiting	
	Pembrolizumab	Advanced Solid Tumors	NCT03486301	1	Recruiting	[121}
BDB018	Pembrolizumab	Advanced Solid Tumors	NCT04840394	1	Recruiting	
BDC-1001	Pembrolizumab	Advanced HER2-Expressing Solid Tumors	NCT04278144	1/2	Recruiting	[122]
LHC-165	Spartalizumab	Advanced Solid Tumors	NCT03301896	1	Active, not recruiting	
SHR-2150	PD-1 mAb	Unresectable/Metastatic Solid Tumors	NCT04588324	2	Recruiting	
BNT411	Atezolizumab	Solid Tumor, Extensive-stage SCLC	NCT04101357	1/2	Recruiting	
Imiquimod	Pembrolizumab	Stage IIIB-IV Melanoma	NCT03276832	1	Recruiting	
DSP-0509	Pembrolizumab	Advanced Solid Tumors	NCT03416335	1/2	Recruiting	
Motolimod	Nivolumab	HNSCC	NCT04272333	1	Recruiting	
			NCT03906526	1	Recruiting	
TLR9						
IMO-2125	Nivolumab and Ipilimumab	Advanced Cancer	NCT04270864	1	Active, not recruiting	
	Nivolumab and Ipilimumab	Solid Tumors	NCT03865082	2	Recruiting	
	Ipilimumab or Pembrolizumab	Metastatic Melanoma	NCT02644967	1/2	Completed	[123]
	Ipilimumab	Anti-PD-1 Refractory Melanoma	NCT03445533	3	Active, not recruiting	[124]
SD-101	Pembrolizumab	Metastatic Melanoma or Recurrent or Metastatic HNSCC	NCT02521870	1/2	Terminated	[126]
	Pembrolizumab	Hormone-Naïve Oligometastatic Prostate Cancer	NCT03007732	2	Recruiting	
	Nivolumab or Ipilimumab	Metastatic Uveal Melanoma	NCT04935229	1	Recruiting	
	Nivolumab	Chemotherapy-Refractory Metastatic Pancreatic Cancer	NCT04050085	1	Recruiting	
	Ipilimumab	Recurrent Low Grade B Cell Lymphoma	NCT02254772	1/2	Completed	[127]
CMP-001	Pembrolizumab	Recurrent or Metastatic HNSCC	NCT04633278	2	Recruiting	
	Pembrolizumab	Advanced Melanoma	NCT03084640	1	Completed	[129]
	Pembrolizumab	Relapsed and Refractory Lymphoma	NCT03983668	1/2	Recruiting	
	Pembrolizumab	Melanoma	NCT02680184	1	Active, not recruiting	
	Pembrolizumab	Patients With Operable Melanoma	NCT04708418	2	Recruiting	
	Nivolumab	Melanoma	NCT04401995	2	Recruiting	
	Nivolumab	Advanced Melanoma	NCT04698187	2	Recruiting	
	Nivolumab	Advanced Melanoma	NCT04695977	2/3	Recruiting	
	Nivolumab	Stage IIIB/C/D Melanoma Patients With Clinically Apparent Lymph Node Disease	NCT03618641	2	Completed	[130]
	Atezolizumab	NSCLC	NCT03438318	1	Completed	
MGN1703	Ipilimumab	Advanced Solid Tumors	NCT02668770	1	Active, not recruiting	

## TLR agonists

Since TLR agonists trigger innate immune cell activation and enhance type I IFNs production in the TME, this is a feasible strategy to synergize with ICBs. As a TLR2/4 agonist, the treatment of BCG combined with ICBs has been regarded as a regimen in clinical trials (Table 1). Interestingly, only anti-PD-1 or anti-PD-L1 antibodies are associated with BCG in these ongoing trials. One trial using BCG followed by ipilimumab treatment in advanced metastatic melanoma reported immune-related adverse events and no evidence of clinical benefits (NCT01838200) [115]. Additionally, these combined trials only studied the effects of BCG in bladder cancer, which is probably due to its FDA approval for bladder cancer. For TLR3 agonists, poly I:C is used to enhance the efficacy of ICBs in preclinical models (Table 1). In an engineered immune cell-poor melanoma mouse model, the targeted activation of the type I IFN system with poly I:C in combination with anti-PD-1 strongly prolonged murine life, thereby suggesting a possible effective strategy to increase the therapeutic efficacy of anti-PD-1/PD-L1 in patients with immune cell-poor melanomas [116]. Similarly, in a study using multiple models of TNBC, poly I:C stimulated PD-L1 expression via type I IFNs, and poly I:C administered in combination with anti-PD-1 treatment was more effective than treatment with anti-PD-1 alone [117]. Moreover, intratumoral administration with BO-112 (nanoplexed poly I:C) reduced MC38, 4T1, and B16-F10 growth in mice, and enhanced the antitumor effect of anti-PD-L1 [118]. Thus, many clinical trials combining TLR3 agonists and ICBs are in progress (Table 1). So far, about 18 TLR7/8 agonists are used in clinical trials for the treatment of cancer and infections [119]. Some of them are combined with ICBs to treat cancers in phase 1/2 (Table 1). BDB001, a TLR 7/8 dual agonist, has been safely administered intravenously to reprogram dendritic cells for antitumor activities [120]. Moreover, intravenously administered BDB001 combined with pembrolizumab has been well-tolerated and resulted in systemic immune activation in a phase 1 dose-escalation trial (NCT03486301) [121]. BDC-1001, a novel TLR 7/8 agonist with HER2 conjugation, has shown immune-mediated antitumor efficacy in preclinical tumor models resistant to anti-HER2 treatments, and its dose escalation by combination with pembrolizumab is ongoing (NCT04278144) [122]. TLR9 agonists have been studied with ICBs in multiple tumor types, including melanoma, lymphoma, head and neck squamous cell carcinoma (HNSCC), and NSCLC (Table 1). Since TLR9 is an intracellular nucleic acid sensor, it has been stimulated with synthetic oligonucleotides (ODN) to activate type I IFN signals. IMO-2125 (Tilsotolimod, commonly referred to as immunomodulatory oligonucleotide (IMO)) is one of the most advanced TLR9 agonists used in clinical trials. In the phase 1/2 trial of PD-1 inhibitor refractory advanced melanoma (NCT02644967), intratumoral IMO-2125 in combination with ipilimumab showed a 71.4% disease control rate and 22.4% overall response rate (ORR) [123]. Based upon these promising results, a phase 3 trial of IMO-2125 in combination with ipilimumab in anti-PD-1 refractory melanoma has been conducted (NCT03445533), but the preliminary results did not meet its primary ORR endpoint [124]. SD-101 is a CpG-ODN with cytidine-phospho-guanosine (CpG) motifs and intratumoral SD-101 overcame PD-1 blockade resistance in mice bearing CT26 tumors [125]. Moreover, SD-101 in combination with pembrolizumab resulted in an ORR of 78% in patients with unresectable or metastatic malignant melanoma (NCT02521870) [126]. However, combining SD-101 with ipilimumab and radiation in patients with recurrent low-grade B cell lymphoma showed as an unpromising therapeutic option (NCT02254772) [127]. Another CpG-ODN TLR9 agonist, CMP-001, enhanced anti-PD-1 therapy in mice-bearing mEERL HNSCC [128] and its combination clinical trial in recurrent or metastatic HNSCC is ongoing (NCT04633278). In addition, a phase 1b study (NCT03084640) of CMP-001 in combination with pembrolizumab had a manageable safety profile and durable response in 25% of patients with metastatic melanoma [129], and further phase 2 trials have been initiated to confirm the efficacy of CMP-001 and nivolumab in advanced melanoma (NCT04401995/NCT04698187/NCT04695977). Furthermore, phase 1/2 trials using CMP-001 and pembrolizumab are ongoing in patients with lymphoma (NCT03983668) and melanoma (NCT02680184/NCT04708418). It is worth noting that the combination of CMP-001 and nivolumab in patients with stage IIIB/C/D melanoma has shown acceptable toxicity and promising efficacy (60% major pathologic response rate (MPR) and 82% 1-year relapse-free survival) [130]. MGN1703, which is DNA-based and essentially different from the CpG-ODN TLR9 agonist, has shown immune activation and anti-tumor efficacy in metastatic solid tumors [131]. However, MGN1703 showed no relevant efficacy in phase 2 trials (NCT02200081) on extensive-stage small-cell lung cancer, even though its favorable safety profile promoted further trials [132]. Further studies found that MGN1703 strengthened the effect of ICBs in preclinical models [133, 134], which supported an ongoing trial combining MGN1703 with ipilimumab in advanced solid tumors (NCT02668770). Thus far, out of all these TLR agonists, TLR9 in combination with ICBs has shown the most encouraging clinical data.

## STING agonists

ICBs, particularly anti­-PD­-1 and anti­-CTLA-4, failed to induce antitumor effects in cGAS or STING­deficient mice when administered alone, which indicated that cGAS-STING signal may need to be screened in patients before STING agonists in combination with ICBs. However, the efficacy of ICBs was substantially increased in tumor models when combined with either the cGAS product, cGAMP, or a synthetic cGAMP analog in cGAS-STING signal sufficient context [37, 135–137]. These results suggest that the combination of ICBs with treatments that aim at the cGAS-STING axis could be an effective strategy to overcome immunosuppression and increase patient responsiveness. Therefore, clinical trials combining STING agonists with ICBs are underway [87]. We summarized these clinical trials in Table 2. Reports found that intratumoral low-dose ADU-S100 (MIW815), one of the synthetic CDNs for STING activation, induced local activation of tumor-specific CD8^+^ T cells for durable anti-tumor immunity and their combination with ICBs resulted in better anti-tumor effects in a poorly immunogenic tumor model [138]. Moreover, intraperitoneal administration of ADU-S100 in colon cancer suppressed aberrant angiogenesis and resulted in an inflamed TME in a type I IFN-dependent manner. Consequently, the combination of ADU-S100 and anti-PD-1 antibody further enhanced the antitumor effect [139]. These preclinical findings supported the clinical trials. In a phase 1b study of PD-1-naive TNBC and PD-1-relapsed/refractory melanoma, the combination of ADU-S100 with spartalizumab was well-tolerated and demonstrated antitumor activity [140]. Furthermore, trials combining ADU-S100 with ipilimumab or pembrolizumab have been initiated (NCT02675439/NCT03937141). The preliminary data of MK-1454, another CDN, alone or in combination with pembrolizumab in patients with advanced solid tumors or lymphomas, resulted in encouraging efficacy and an acceptable safety profile (NCT03010176), which makes us look forward to the final result [141]. In addition, intratumoral MK-1454 in combination with pembrolizumab to treat metastatic or unresectable, recurrent HNSCC is being tested in phase 2 (NCT04220866). In addition, to increase STING-dependent type I IFN production, researchers have engineered CDNs with cancer vaccines to form the STINGVAX. Interestingly, PD-L1 expression in tumor cells from STINGVAX-treated mice was significantly upregulated. Thus, combining PD-1 blockade with STINGVAX increased the anti-tumor efficacy in many tumor models that did not respond to anti-PD-1 alone, which supports the rationale for clinical evaluation of STINGVAX in combination with anti-PD-1, particularly in settings where patients failed to respond to ICB monotherapy [135]. Moreover, using non-pathogenic *E coli Nissle*, researchers designed SYNB1891, an agonist for targeting STING, to induce anti-tumor immunity and immunological memory [142]. In addition, a phase 1 study of SYNB1891 injection alone or in combination with atezolizumab is being tested in advanced/metastatic solid tumors and lymphoma (NCT04167137). Since the STING signal was discovered after TLR, clinical trials using STING agonists in combination with ICBs are mostly in phase 1 (Table 2).

**Table 2:** STING agonists in combination with ICBs

STING agonist	ICB	Cancer type	Trial NO	Phase	Status	Refs.
ADU-S100	Ipilimumab	Advanced Solid Tumors or Lymphomas	NCT02675439	1	Active, not recruiting	
	Spartalizumab	Advanced Solid Tumors or Lymphomas	NCT03172936	1	Completed	[140]
	Pembrolizumab	Metastatic or Recurrent HNSCC	NCT03937141	2	Active, not recruiting	
MK-1454	Pembrolizumab	Advanced/Metastatic Solid Tumors or Lymphomas	NCT03010176	1	Active, not recruiting	[141]
		Metastatic or Unresectable, Recurrent HNSCC	NCT04220866	2	Active, not recruiting	
SYNB1891	Atezolizumab	Advanced/Metastatic Solid Tumors or Lymphomas	NCT04167137	1	Recruiting	
MK-2118	Pembrolizumab	Advanced/Metastatic Solid Tumors or Lymphomas	NCT03249792	1	Recruiting	
GSK3745417	Dostarlimab	Advanced Solid Tumors	NCT03843359	1	Recruiting	
SNX281	Pembrolizumab	Advanced Solid Tumors and Lymphoma	NCT04609579	1	Recruiting	
BMS-986301	Nivolumab or Ipilimumab	Advanced Solid Tumors	NCT03956680	1	Recruiting	
SB11285	Atezolizumab	Advanced Solid Tumors	NCT04096638	1	Recruiting	

## Radiation therapy

RT has been demonstrated as a promising strategy to induce type I IFN secretion in the TME. RT is a well-established, accessible, and comparatively economical procedure that has been widely used in combination with ICBs in clinical trials, owing to its cytotoxic, and multiple immunomodulatory effects on tumor cells as well as its relatively limited and manageable clinical side effects [143–145]. Multiple phase 1/2 clinical trials that combined RT with ICBs have acquired promising results and smoothly implemented phase 3 clinical trials [144]. Among these, durvalumab improved the progression-free survival and overall survival rate in stage III unresectable NSCLLC after chemoradiotherapy (NCT02125461) [146]. In addition, ongoing phase 3 trials are using durvalumab in combination with chemoradiation in patients with locally advanced cervical cancer (NCT03830866) [147] and limited-stage small-cell lung cancer (NCT03703297) [148]. However, ipilimumab had no significant effect in terms of overall survival compared to the placebo after RT in a multicenter, randomized, double-blind, phase 3 trial (NCT00861614) in patients with metastatic castration-resistant prostate cancer [149]. Moreover, in two phase 3 glioblastoma trials (NCT02667587/NCT02617589), nivolumab plus RT did not meet the primary endpoint of overall survival [150]. Despite some disappointing clinical survival benefit results, trials combining RT with ICBs are ongoing in multiple tumors. We summarized the present combination regimens of RT with ICBs in phase 3 trials in Table 3. ICBs may be a feasible option in patients where RT failed, but the disappointing data in some of the phase 3 trials suggested that we need to broaden our understanding of the underlying molecular, cellular, and systemic mechanisms of these combination treatments. Recently, researchers found that RT induced the upregulation of PD-L1 and TGF-β, which can be blocked by bintrafusp alfa, a bifunctional fusion protein targeting both PD-L1 and TGF-β. Moreover, bintrafusp alfa in combination with RT eradicated therapy-resistant tumors [151]. This study indicated that involving a specific target for RT in combination with ICBs may be more effective in specific patients, especially in RT-induced immunosuppressive molecules.

**Table 3:** Radiaton therapy in combination with ICBs in Phase 3/4

ICB	Cancer type	Trial NO	Status	Refs.	RT schedule
Ipilimumab	Advanced Prostate Cancer	NCT00861614	Completed	[149]	
Nivolumab	Human papillomavirus (HPV) Throat Cancer	NCT03811015	Recruiting		IMRT 35 fractions
	Early-Stage, HPV-Positive, Non-Smoking Associated Oropharyngeal Cancer	NCT03952585	Recruiting		IMRT/IGRT 36 fractions
	HNSCC	NCT03576417	Recruiting		IMRT 66 Gy/6.5 weeks
	Cisplatin-ineligible or Eligible Locally Advanced HNSCC	NCT03349710	Completed		
	Newly Diagnosed Patients With Glioblastoma	NCT02667587	Active, not recruiting	[150]	RT 60 Gy/6 weeks
	Newly Diagnosed Patients With Glioblastoma	NCT02617589	Active, not recruiting	[150]	
Ipilimumab and Nivolumab	Stage IV NSCLC	NCT03391869	Recruiting		
	Esophageal and Gastroesophageal Junction Adenocarcinoma Undergoing Surgery	NCT03604991	Recruiting		
	Newly Diagnosed Tumor O-6-Methylguanine DNA Methyltransferase Unmethylated Glioblastoma	NCT04396860	Recruiting		RT 30 fractions/6 weeks
Pembrolizumab	Newly Diagnosed Endometrial Cancer After Surgery With Curative Intent	NCT04634877	Recruiting		EBRT > or = 4500 cGy
	Newly Diagnosed Early-Stage High Intermediate Risk Endometrial Cancer	NCT04214067	Recruiting		
	Unresected Stage I or II NSCLC	NCT03924869	Recruiting		SBRT 45–70 Gy/2 weeks
	Stage IV NSCLC	NCT03867175	Recruiting		
	Advanced NSCLC	NCT03774732	Recruiting		3D-CRT/SABR 18 Gy
	Advanced HNSCC	NCT03765918	Recruiting		RT 60–70 Gy/30–35 fractions
	Newly Diagnosed Metastatic HNSCC	NCT04747054	Recruiting		Loco-regional RT 54 Gy/18 fractions
	Completely Resected Stage I-III Merkel Cell Cancer	NCT03712605	Recruiting		
	Muscle-invasive Bladder Cancer	NCT04241185	Recruiting		RT 55–64 Gy/4–6.5 fractions
	Advanced Esophageal Squamous Cell Carcinoma	NCT04807673	Recruiting		RT 41.4 Gy/23 fractions
Atezolizumab	Early NSCLC	NCT04214262	Recruiting		
	Extensive Stage SCLC	NCT04402788	Recruiting		
	Limited Stage SCLC	NCT03811002	Recruiting		
	High-Risk HNSCC	NCT01810913	Recruiting		
Durvalumab	Stage III Unresectable NSCLC	NCT04613284	Not yet recruiting		3DCRT/IMRT 50 Gy
	Stage III Unresectable NSCLC	NCT04597671	Recruiting		Low-dose PCI 15 Gy/10fractions
	Early-Stage Unresected NSCLC	NCT03833154	Recruiting		SBRT 3–8 fractions
	Unresectable NSCLC	NCT03519971	Active, not recruiting		
	Locally Advanced Cervical Cancer	NCT03830866	Active, not recruiting	[147]	
	Stage III Unresectable NSCLC	NCT02125461	Active, not recruiting	[146]	
	Limited Stage SCLC	NCT03703297	Active, not recruiting	[148]	RT 45 Gy/3 weeks or 60–66 Gy/6 weeks
Avelumab	HNSCC	NCT02999087	Active, not recruiting		IMRT 69.96 Gy or 52.8 Gy/6.5 weeks

## Oncolytic virotherapy

Currently, few OVs have been officially approved for use in clinical settings. However, combinations of various OVs with ICBs are currently being tested in clinical trials, including HSV-1/2, Adenovirus (Ad), Vaccinia virus (VV), Coxsackievirus; Polio/rhinovirus, Maraba virus (MRB), Vesicular stomatitis virus (VSV), and Reovirus (Table 4). T-VEC has gained a durable response in melanoma [152] and its combination with ipilimumab in unresected melanoma has shown greater antitumor activity without additional safety concerns compared to monotherapy (NCT01740297) [153]. In addition to its combination with anti-CTLA-4, T-VEC combined with pembrolizumab increased IFN-γ and CD8^+^ T cells in patients with advanced melanoma [154]. For most trials using T-VEC plus ICBs, phase 1 has completed and phase 2 will likely soon be initiated (Table 4). Moreover, the combination of T-VEC with pembrolizumab has shown promising ORR and CR rates in advanced melanoma (NCT02263508) [155]. However, in a multicenter phase 1b study, the combination of T-VEC and pembrolizumab has shown a tolerable safety profile in recurrent or metastatic HNSCC, but its efficacy was similar to that of pembrolizumab monotherapy [156]. Thus, this combination regimen will not be continued to phase 3. HF10, another HSV-1-derived OV, is well-tolerated and has continued viral antitumor activity in refractory and superficial cancers [157]. Furthermore, the combination of HF10 and ipilimumab showed favorable profiles in two phase 2 studies of patients with unresectable or metastatic melanoma (NCT02272855/NCT03153085) [158, 159]. Other HSV-1-derived OVs in combination with ICBs are being tested in phase 1/2 trials (Table 4). In preclinical studies, both NDV and Maraba-based OVs induced increased PD-L1 expression in melanoma [107] and breast cancer [105]. Therefore, the combination of OVs with ICBs is a promising strategy to overcome PD-L1-induced immunotherapy resistance. Zamarin et al. showed that localized oncolytic NDV in combination with systemic anti-CTLA-4 blockade can eradicate tumors in B16 melanoma by producing curative immune responses that require CD8^+^ T cells, NK cells, and type I IFNs [102]. Therefore, MEDI9253 (NDV human IL-12) in combination with durvalumab is being used in an ongoing phase 1 trial (NCT04613492). Ad is another large class of OVs, and many of them are combined with ICBs in phase 1/2 trials (Table 4). ONCOS-102, an Ad-based OV that expresses GM-CSF, in combination with pembrolizumab reduced tumor volume in a humanized A2058 melanoma mouse model that did not benefit from pembrolizumab monotherapy [160]. Moreover, a phase 1 trial of ONCOS-102 plus pembrolizumab has shown a 33% ORR in advanced or unresectable melanoma progressing after PD-1 blockade (NCT03003676) [161]. These promising data promoted the use of other Ads in combination with ICBs in trials (Table 4). MRB-based OVs are used in ongoing trials as well, such as MG1-MAGEA3 and MG1-E6E7. To expand more tumor-associated antigens in the TME, two trials used MRB and Ad-based OVs (MG1-MAGEA3 and Ad-MAGEA3; MG1-E6E7 and Ad-E6E7) in combination with ICBs for NSCLC or HPV-associated cancers (NCT02879760/ NCT03618953). The use of Coxsackievirus A21 (CVA21), a naturally occurring OV, in combination with ICBs in a phase 1 trial has shown a well-tolerated and durable response (NCT02307149/NCT02565992) [162, 163]. Moreover, phase 2 trials using CVA21 in combination with pembrolizumab are recruiting participants (Table 4). Recently, researchers found that monotherapy with Measles virus-based neutrophil-activating protein or anti-PD-1 treatment has shown a modest survival benefit in Measles virus-resistant syngeneic glioblastoma models, and that combination treatment had a synergic effect [164]. Other OVs, such as PVSRIPO, VSV-IFNβ-NIS, Pelareorep (Reovirus), and OH2 (HSV-2), in combination with ICBs are in trials as well. In general, the use of OVs plus ICBs is a promising regimen, especially T-VEC plus pembrolizumab in patients with melanoma, which is currently in phase 3.

**Table 4:** Oncolytic virus in combination with ICBs

Oncolytic virus	ICB	Cancer type	Trial NO	Phase	Status	Refs.
HSV 1						
T-VEC	Atezolizumab	Early Breast Cancer	NCT03802604	1	Recruiting	
	Atezolizumab	Triple-Negative Breast Cancer and Colorectal Cancer With Liver Metastases	NCT03256344	1	Active, not recruiting	
	Pembrolizumab	Recurrent Metastatic HNSCC	NCT02626000	1	Completed	[156]
	Ipilimumab	Unresected Melanoma	NCT01740297	1/2	Completed	[153]
	Pembrolizumab	Liver Tumors	NCT02509507	1/2	Active, not recruiting	
	Nivolumab and Ipilimumab	Localized, Triple-Negative or Estrogen Receptor Positive, HER2 Negative Breast Cancer-deleted	NCT04185311	1	Active, not recruiting	
	Nivolumab	Refractory Lymphomas or Advanced or Refractory Non-melanoma Skin Cancers	NCT02978625	2	Recruiting	
	Pembrolizumab	Metastatic and/or Locally Advanced Sarcoma	NCT03069378	2	Recruiting	
	Pembrolizumab	Stage III-IV Melanoma	NCT02965716	2	Active, not recruiting	
	Pembrolizumab	Stage III Melanoma	NCT03842943	2	Recruiting	
	Pembrolizumab	Unresectable/Metastatic Melanoma	NCT04068181	2	Active, not recruiting	
	Pembrolizumab	Unresected Melanoma	NCT02263508	3	Terminated	[155]
HF10	Ipilimumab	Unresectable or Metastatic Melanoma	NCT02272855	2	Completed	[158]
	Ipilimumab	Unresectable or Metastatic Melanoma	NCT03153085	2	Completed	[159]
T3011 (Intratumoral)	Pembrolizumab	Advanced or Metastatic Solid Tumors	NCT04370587	1/2	Recruiting	
T3011 (Intravenous)	Pembrolizumab	Advanced or Metastatic Solid Tumors	NCT04780217	1/2	Recruiting	
ONCR-177	Pembrolizumab	Advanced and/or Refractory Cutaneous, Subcutaneous or Metastatic Nodal Solid Tumors or With Liver Metastases of Solid Tumors	NCT04348916	1	Recruiting	
RP1	Cemiplimab	Advanced Squamous Skin Cancer	NCT04050436	2	Recruiting	
	Nivolumab	Advanced and/or Refractory Solid Tumors	NCT03767348	2	Recruiting	
Ad						
ONCOS-102	Pembrolizumab	Advanced or Unresectable Melanoma Progressing After PD-1 Blockade	NCT03003676	1	Completed	[161]
	Durvalumab	Advanced Peritoneal Malignancies	NCT02963831	1/2	Active, not recruiting	
LOAd703	Atezolizumab	Pancreatic Cancer	NCT02705196	1/2	Recruiting	
		Malignant Melanoma	NCT04123470	1/2	Recruiting	
Adenovirus CCL21	Pembrolizumab	Stage IV NSCLC	NCT03546361	1	Recruiting	
NG-641	Nivolumab	Metastatic or Advanced Epithelial Tumors	NCT05043714	1	Not yet recruiting	
ADV/HSV-tk	Pembrolizumab	Metastatic NSCLC, Metastatic Triple-negative Breast Cancer	NCT03004183	2	Active, not recruiting	
ChAdOx1-MVA 5T4	Nivolumab	Advanced Prostate Cancer	NCT03815942	1/2	Active, not recruiting	
DNX-2401	Pembrolizumab	Recurrent Glioblastoma or Gliosarcoma	NCT02798406	2	Completed	
VB-111	Nivolumab	Metastatic Colorectal Cancer	NCT04166383	2	Recruiting	
Adenoviral-mediated IL-12	Pembrolizumab	Triple-Negative Breast Cancer	NCT04095689	2	Recruiting	
VCN-01	Durvalumab	HNSCC	NCT03799744	1	Recruiting	
SynOV1.1	Atezolizumab	Hepatocellular Carcinoma	NCT04612504	1/2	Not yet recruiting	
OBP-301	Pembrolizumab	HNSCC With Inoperable Recurrent or Progressive Disease	NCT04685499	2	Recruiting	
VV						
TBio-6517	Pembrolizumab	Advanced Solid Tumors	NCT04301011	1/2	Recruiting	
P53MVA	Pembrolizumab	Recurrent Ovarian, Primary Peritoneal, or Fallopian Tube Cancer	NCT03113487	2	Recruiting	
	Pembrolizumab	Solid Tumors That Have Failed Prior Therapy	NCT02432963	1	Active, not recruiting	
Pexa-Vec (JX-594)	Durvalumab and Tremelimumab	Refractory Colorectal Cancer	NCT03206073	1/2	Active, not recruiting	
	Ipilimumab	Metastatic / Advanced Solid Tumors	NCT02977156	1	Recruiting	
	Cemiplimab	Renal Cell Carcinoma	NCT03294083	1/2	Recruiting	
BT-001	Pembrolizumab	Metastatic or Advanced Solid Tumors	NCT04725331	1/2	Recruiting	
PROSTVAC	Nivolumab	Prostate Cancer	NCT02933255	1/2	Recruiting	
	Nivolumab and Ipilimumab	Metastatic Hormone-Sensitive Prostate Cancer	NCT03532217	1	Active, not recruiting	
CV301	Nivolumab	Metastatic Colorectal Cancer	NCT03547999	2	Active, not recruiting	
TG4010	Nivolumab	NSCLC	NCT02823990	2	Active, not recruiting	
Coxsackievirus						
CVA21	Ipilimumab	Advanced Melanoma	NCT02307149	1	Completed	[162]
	Pembrolizumab	Advanced Melanoma	NCT02565992	1	Completed	[163]
	Ipilimumab	Uveal Melanoma Metastatic to the Liver	NCT03408587	1	Completed	
	Pembrolizumab	NSCLC and Bladder Cancer	NCT02043665	1	Completed	
	Pembrolizumab	Advanced/Metastatic Solid Tumors	NCT04521621	1/2	Recruiting	
	Pembrolizumab	Stage III Melanoma	NCT04303169	1/2	Recruiting	
	Pembrolizumab	Advanced/Metastatic Melanoma	NCT04152863	2	Recruiting	
Polio/rhinovirus						
PVSRIPO	PD-1 mAb	Advanced PD-1 Refractory Melanoma	NCT04577807	2	Recruiting	
	PD-1/L1 mAb	Advanced Solid Tumors	NCT04690699	1/2	Recruiting	
	Pembrolizumab	Recurrent Glioblastoma	NCT04479241	2	Recruiting	
MRB						
MG1-MAGEA3 and Ad-MAGEA3	Pembrolizumab	NSCLC	NCT02879760	1/2	Completed	
MG1-E6E7 and Ad-E6E7	Atezolizumab	HPV Associated Cancers	NCT03618953	1	Active, not recruiting	
VSV						
VSV-IFNβ-NIS	Pembrolizumab	NSCLC and HNSCC	NCT03647163	1/2	Recruiting	
	Avelumab	Malignant Solid Tumor	NCT02923466	1	Active, not recruiting	
Reovirus						
Pelareorep	Nivolumab	Relapsed/Refractory Multiple Myeloma	NCT03605719	1	Recruiting	
	Atezolizumab	Early Breast Cancer	NCT04102618	1	Recruiting	
	Avelumab	Metastatic Breast Cancer	NCT04215146	2	Recruiting	
NDV						
MEDI9253	Durvalumab	Solid Tumors	NCT04613492	1	Recruiting	
HSV 2						
OH2	Pembrolizumab	Advanced Solid Tumors	NCT04386967	1/2	Recruiting	

## Others

Currently, many additional curative combinatorial agents targeting the type I IFN signaling axis are being developed. For instance, based on the characteristic of Mn to induce type I IFN production, the combination of Mn and anti-PD-1 antibody has been used in a phase 1 study of patients with advanced metastatic solid tumors (NCT03991559), which showed manageable safety and promising efficacy [25]. In addition, several phase 2 studies using a combined regimen of Mn and anti-PD-1 antibody in advanced solid tumors or lymphoma are ongoing (NCT04004234/ NCT03989310/NCT03989336/NCT04873440). The nucleoside analog 6-thio-2’-deoxyguanosine (6-thio-dG), which induces damage to telomeric DNA in telomerase-expressing tumor cells to initiate the host cytosolic DNA-sensing type I IFNs/STING pathway, was reported to overcome ICB resistance in advanced tumors and was used in combination with ICBs in tumor models [165]. Moreover, oral delivery of live *Lactobacillus rhamnosus* GG (LGG), one of the most well-characterized and used probiotics, synergizes with anti-PD-1 to augment antitumor immunity. Combination therapy of LGG and anti-PD-1 mechanically increases tumor-infiltrating DCs and IFN-β induction through the cGAS-STING-IRF7 cascade [166].

## Conclusions and perspectives

Type I IFNs were first described for their strong antiviral properties, and mounting evidence confirmed their anti-tumor activity. To date, IFN-α and IFN-β have achieved some beneficial results in patients with cancer, and emerging clinical data support the key role of type I IFN inducers in combination with ICBs. These antitumor effects originate from type I IFNs produced by tumor cells or immune effector cells in response to pathogenic molecular stimuli. In turn, type I IFNs exert anti-tumor functions by directly influencing tumor cell progression and indirectly modulating anti-tumor immune cells in multiple ways. Nevertheless, large gaps in our understanding of the mechanism by which substances and signals are transferred between tumor cells, type I IFN, and immune cells remain. Thus, further elucidating the reciprocal interactions among tumor cells, type I IFN, and immune cells will be of interest for future studies. Furthermore, the cell population of type I IFN-produced cells, such as DC and endothelial cells, is limited in the TME; thus, the baseline of type I IFNs is lower. This prompts researchers to find the regulatory mechanism of type I IFNs. A more in-depth appreciation of the molecular signaling cascades and the series of genes activated by type I IFNs will provide multiple opportunities for enhancing IFN-I-based tumor therapy.

To overcome the lower baseline level, many type I IFN inducers were used to treat tumors in preclinical models and clinical trials. However, its anti-tumor effect is unsatisfactory in certain circumstances, which may be due to recombinant type I IFNs causing systemic side effects or certain type I IFN inducers causing the increased production of immunosuppressive molecules, such as ROS from neutrophil, PD-L1 in tumor cells, and IDO in immune cells. In addition, the specific tumor background should be considered when performing the therapeutic schedule. However, intratumoral injection or nanoparticle-packaged type I IFN inducers have been used in trials to reduce these side effects. Furthermore, to neutralize immunosuppressive molecules, type I IFN inducers in combination with ICB, IDO, and other specific inhibitors may overcome this resistance.

Many clinical trials that combine type I IFN inducers with ICBs are in progress. Some final results of phase 3 studies did not meet the primary endpoints; thus, the essential mechanism of resistance caused by ICB or type I IFN inducer monotherapy needs to be clarified. These studies also suggest that not all patients are suitable for combination therapy of type I IFN inducers and ICBs. The addition of type I IFN signaling as an efficacy prediction factor of ICB may promote the precisive application of type I IFN inducers and ICBs in future. Therefore, we look forward to improved clinical effects in these ongoing trials.

## Data Availability

Not applicable.

## Abbreviations

ICB: Immune checkpoint blockade
IFNs: Interferons
IFNAR1: IFN-α/β receptor 1
IFNAR2: IFN-α/β receptor 2
TYK2: Tyrosine kinase 2
JAK1: Janus kinase 1
STAT1: Signal transducer and activator of transcription 1
STAT2: Signal transducer and activator of transcription 2
IRF9: Interferon regulatory factor 9
ISGF3: IFN-stimulated gene factor 3
ISREs: IFN-stimulated response elements
ISGs: Interferon-stimulated genes
MAPK: Mitogen-activated protein kinase
mTOR: Mammalian target of rapamycin
GCD: GCD-GTPases/cyclin-dependent kinases
FDA: Food and Drug Administration
TME: Tumor microenvironment
DCs: Dendritic cells
pDCs: Plasmacytoid dendritic cells
CD: Cluster of differentiation
GMP: Guanosine monophosphate
AMP: Adenosine monophosphate
cGAMP: Cyclic GMP–AMP
cGAS: CGAMP synthase
STING: Stimulator of interferon genes
ER: Endoplasmic reticulum
TBK1: TANK-binding kinase 1
IRF: Interferon regulatory factor
NF-κB: Nuclear factor-kappa B
Mn: Manganese
dsDNA: Double-stranded DNA
HMGB1: High-mobility group box 1
TLR: Toll-like receptor
MyD88: Myeloid differentiation factor 88
CLEC9A: C-type lectin domain-containing 9A
SLC19A1: Solute Carrier Family 19 Member 1
RIG-I: Retinoic acid­inducible gene I
RLRs: RIG-I-like receptors
PRRs: Pattern recognition receptors
UNC93B1: Unc-93 homolog B1
PTX: Paclitaxel
TICAM1: Toll-like receptor adaptor molecule 1
LPS: Lipopolysaccharide
TAMs: Tumor-associated macrophages
E-FABP: Epidermal fatty acid-binding proteins
LD: Lipid droplet
CAFs: Cancer-associated fibroblasts
ZBP1: Z-DNA binding protein 1
DDX41: DEAD-Box Helicase 41
MHC I: Major histocompatibility complex class I
TAA: Tumor-associated antigen
APC: Antigen-presenting cells
NK: Natural killer
IL: Interleukin
CXCR: C-X-C chemokine receptor
CXCL: C-X-C chemokine ligand
ROS: Reactive oxygen species
Bcl-2: B cell lymphoma-2
BCL-xL: B cell lymphoma-extra-large
G-CSF: Granulocyte colony-stimulating factor
VEGF: Vascular endothelial growth factor
MMP9: Matrix metallopeptidase 9
Treg: Regulatory T cells
MEK: Mitogen-activated protein kinase kinase
ERK: Extracellular signal-regulated kinase
PDE4: Phosphodiesterase 4
cAMP: Cyclic AMP
CCL: C–C chemokine ligand
MDSCs: Myeloid-derived suppressor cells
TRAIL: Tumor necrosis factor (TNF)-related apoptosis-inducing ligand
DR5: Death receptor 5
IDO1: Indoleamine 2,3-dioxygenase 1
Poly A:U: Polyadenylic–polyuridylic acid
Poly(ICLC): Poly I:C plus polylysine
CpG: Cytidine-phospho-guanosine
BCG: Bacillus Calmette–Guérin
CDNs: Cyclic dinucleotides
CAR T cell: Chimeric antigen receptor T cell
NSCLC: Non-small-cell lung cancer
cGAMP-NPs: CGAMP nanoparticles
RT: Radiation therapy
OVs: Oncolytic viruses
HER2/ErbB-2: Human epidermal growth factor receptor 2
Anti-CSF-1R: Anti-colony-stimulating factor-1 receptor
ATM: Ataxia-telangiectasia-mutated
ATRi: ATM and Rad3-related protein kinase inhibitor
T-VEC: Talimogene laherparepvec
HSV: Herpes simplex virus
GM-CSF: Granulocyte–macrophage colony-stimulating factor
NDV: Newcastle disease virus
IPS-1: Interferon-beta promoter stimulator 1
Trif: Toll/IL-1R domain-containing adaptor inducing IFN-β factor
iPSC: Induced pluripotent stem cell
iPSC-pMC: IPSC-derived proliferating myeloid cell
XCR1: X-C Motif Chemokine Receptor 1
PD-1: Programmed cell death 1
PD-L1: Programmed death ligand 1
ODN: Oligonucleotides
IMO: Immunomodulatory oligonucleotide
ORR: Objective response rate
HNSCC: Head and neck squamous cell carcinoma
MPR: Major pathologic response rate
CTLA-4: Cytotoxic lymphocyte-associated antigen-4
TNBC: Triple-negative breast cancer
TGF-β: Transforming growth factor beta
Ad: Adenovirus
VV: Vaccinia virus
MRB: Maraba virus
VSV: Vesicular stomatitis virus
CR: Complete response
CVA21: Coxsackievirus A21
6-thio-dG: 6-Thio-20-deoxyguanosine
LGG: *Lactobacillus rhamnosus* GG
IMRT: Intensity-modulated radiation therapy
IGRT: Image guided radiation therapy
EBRT: External beam radiotherapy
SBRT: Stereotactic body radiotherapy
3D-CRT: 3-Dimensional conformal radiation therapy
SABR: Stereotactic ablative radiotherapy
PCI: Prophylactic cranial irradiation
